# Semen preparation methods and sperm telomere length: density gradient centrifugation versus the swim up procedure

**DOI:** 10.1038/srep39051

**Published:** 2016-12-13

**Authors:** Feifei Zhao, Qingling Yang, Senlin Shi, Xiaoyan Luo, Yingpu Sun

**Affiliations:** 1 Reproductive Medical Center, First Affiliated Hospital of Zhengzhou University, China

## Abstract

Previous studies have shown that both density gradient centrifugation (DGC) and swim up (SU) procedures can select spermatozoa with longer telomeres for assisted reproduction techniques (ART). However, it is unknown which approach is more effective. The aim of the present study was to compare the effects of these two methods on sperm telomere length (STL). A total of 150 normozoospermic subjects were recruited. STL, DNA fragmentation index (DFI), reactive oxygen species (ROS) content and progressive motility of semen samples were detected before and after the procedures of DGC and SU. When compared to raw semen, the average length of sperm telomeres was significantly longer after the two sperm preparation methods. However, no significant difference was found between the DGC and SU procedures. We also found that semen prepared by the two methods had lower DNA fragmentation, ROS content and sperm progressive motility. However, no significant difference was found in those parameters between the two procedures. This is the first study that compares the effects of the DGC and SU procedures on STL, and the results show that both methods can recover a sperm population with longer STL and better DNA integrity for ART.

DNA integrity is crucial for the functions of germ cells and the development of early embryos. Many studies have shown that damage to sperm DNA can contribute to the failure of assisted reproductive technologies (ART). Sperm DNA damage can ultimately lead to failed fertilization and an increased risk of abnormal embryo development and the early loss of pregnancy after intrauterine insemination (IUI), *in vitro* fertilization (IVF) and intracytoplasmic sperm injection (ICSI)^1^,^2^,^3^,^4^,^5^,^6^,^7^,^8^.

Telomeres consist of tandem repeat sequences and telomere-associated proteins that are located at the ends of the eukaryote chromosomes and maintain the DNA integrity^9^,^10^. Many studies have focused on the role of telomeres in reproduction^11^, and a series of reports have suggested that sperm telomeres have a significant reproductive function. It has been revealed that the sperm telomere length (STL) is shorter in males who are infertile^12^ and that the loss of telomere length results in the aging and apoptosis of male germ cells as well as the arrest of meiosis in female germ cells^13^. What’s more, the sperm with short telomeres derived from the late generation of telomerase-null mice (TR^−/−^) would have some bad influence on the fertilization and embryonic development, More specifically, fertilization of TR^−/−^ sperm with wild-type eggs exhibited low rates of cleavage and development to morula and blastocysts^14^. Moreover, our previous study demonstrated a positive correlation between the telomere length and embryo quality in the early stages of development^15^, and another study reported that spermatozoa telomeres determine the telomere length of early embryos and offspring^16^. A recent report also showed that the STL could be used as a new parameter in diagnosing infertility in males^17^. Therefore, the selection of functional sperm with less DNA damage and longer telomeres should be prerequisites for achieving optimal outcomes for ART.

ART has been regarded as the main treatment for males and females who suffering from infertility in the past decades. Sperm quality plays a prominent role in ART treatment outcomes^18^. Since a variety of methods have been used to select high quality sperm for fertilizing eggs for ART in the past two decades, those methods have had a direct impact on the results of ART^19^. The swim up (SU) and density gradient centrifugation (DGC) procedures, which are gentle and recover a more functional sperm population, are the most common processing methods used in ART laboratories. These two methods have generated much interest in identifying which technique is more efficient. However, most studies have focused on traditional parameters, such as the recovery rate and conventional semen parameters^19^,^20^. In addition, no obvious consensus has been reached on this subject^21^,^22^,^23^,^24^,^25^. Recently, new molecular parameters, such as DNA integrity, are introduced to evaluate the quality and function of the sperm prepared by these techniques. Nevertheless, the results remain discordant^26^,^27^,^28^,^29^,^30^. One study that compared the effects of DNA fragmentation of sperm recovered by DGC and SU procedures, either alone or in combination, found no difference between the techniques^31^. Amiri and colleagues found that the number of DNA-fragmented spermatozoa in samples prepared by the SU method was larger than those in samples processed with the DGC approach^32^. However, some studies have found that the mean DNA fragmentation in samples processed by DGC was higher than in samples collected with the SU method^33^,^34^. Telomeres are fundamental for genome integrity^17^. Santiso *et al*. and our previous study showed that either the SU or DGC method could screen out sperm with longer telomeres and lower DNA fragmentation for fertilization^35^,^36^. However, no study has examined the effects of these two methods on the STL of sperm.

Thus, the aim of the present study was to compare the effect of collecting sperm with the DGC and SU methods on STL and DNA fragmentation. In addition, the reactive oxygen species (ROS) content and other semen parameters were also compared, and we investigated the correlation between the STL and semen parameters in untreated semen samples.

## Results

### The comparison of sperm population parameters before and after semen processing by the DSG and SU methods

**Figure 1:**
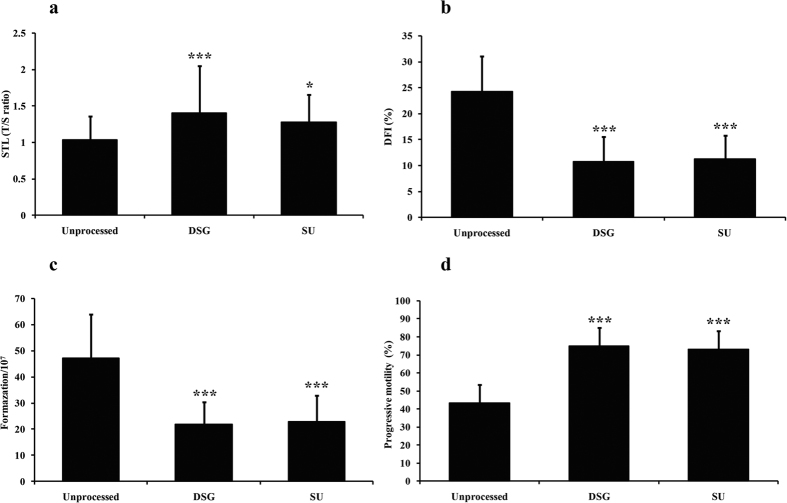
Comparison of the STL, DFI, ROS content and progressive motility before and after the processing methods. (**a**) Comparison of the STL among the three groups. (**b**) Comparison of the DFI among the three groups. (**c**) Comparison of the ROS content among the three groups. (**d**) Comparison of progressive motility among the three groups. *P < 0.05 compared to unprocessed sperm; ***P < 0.001 compared to unprocessed sperm. DFI, DNA fragmentation index. N = 150, One-way ANOVA and *LSD-t* test.

We collected 150 semen samples from 150 normozoospermic men. The age, body mass index (BMI), duration of infertility, and sexual abstinence of these males and the basic seminal parameters are shown in Table 1. The STL, DFI, ROS content and progressive motility before and after the semen was processed by the DGC and SU methods were compared (Fig. 1). Figure 1a shows that the STL was significantly longer after the samples were prepared by the DGC (Fig. 1a, *P* < 0.001) and SU methods (Fig. 1a, *P* = 0.011), but no significant difference was observed between two methods (Fig. 1a, *P* = 0.18). The DFI, which is associated with the development of early embryos and the outcomes of ART, was also analyzed. As expected, the DFI significantly declined in the samples after they were prepared by the DGC (Fig. 1b, *P* < 0.001) and SU methods (Fig. 1b, *P* < 0.001); however, we did not found the significant difference between two ways (Fig. 1b, *P* = 0.66).

It is well-documented that oxidative stress is closely related to the DNA damage and telomere length^10^; therefore, the ROS content was determined before and after the samples were treated by these two preparation methods. No significant difference in the ROS level was found between the DGC-prepared group and the SU-prepared group (Fig. 1c, *P* = 0.66), but the ROS content of semen samples was significantly decreased after pre-processing by DGC (Fig. 1c, *P* < 0.001) and the SU method (Fig. 1c, *P* < 0.001). In addition, the progressive motility of semen samples among three groups was also analyzed. The mean progressive motility of semen samples was significantly higher after the samples were prepared by DGC (Fig. 1d, *P* < 0.001) and the SU method (Fig. 1d, *P* < 0.001). However, the mean progressive motility of the semen samples prepared by the two processing methods exhibited no significant difference (Fig. 1d, *P* = 0.35).

### The correlation between the STL and other semen parameters

A correlation analysis was also performed between the STL and the patient age, duration of infertility, sexual abstinence, sperm volume, sperm concentration, normal sperm morphology, total sperm count, progressive motility, DNA fragmentation, and ROS content. The results are shown in Table 2. The STL was significantly and positively associated with the total sperm count (Table 2, *P* = 0.002) as well as progressive motility (Table 2, *P* = 0.003). However, the STL was significantly and negatively associated with sperm DFI (Table 2, *P* = 0.002) and ROS content (Table 2, *P* = 0.004). In addition, no significant association was observed between the STL and patient age (Table 2, *P* = 0.46) and other parameters respectively (Table 2).

## Discussion

Sperm quality plays a prominent role in achieving a pregnancy through ART, and a variety of procedures exist for selecting better quality sperm for egg fertilization^18^. However, the choice of procedures is very subjective^37^,^38^. Although some procedures are widely used in this field, no consensus exists regarding which method is more suitable. Comparison studies have focused on conventional semen analysis instead of sperm DNA status^20^. Sperm DNA integrity is essential for fertilization and the development of embryos^7^. Telomere length and DNA fragmentation represent the status of DNA integrity^10^. An increasing number of studies have shown that the STL plays a significant role in human reproduction. In meiosis, telomere length guarantees the synapsis, recombination and segregation of homologs^39^. Moreover, the migration of sperm telomeres during spermatogenesis plays a key role in fertilization and pronucleus formation, and a positive correlation exists between the STL and the embryo quality in the early stage^40^. Many studies have demonstrated that high levels of sperm DNA fragmentation may have a negative influence on the embryo morphology, blastocyst formation and implantation rate after IVF and ICSI cycles^6^,^7^,^8^. Therefore, it is especially important for sperm preparation to recover a sperm population with longer telomere length and lower DNA fragmentation.

DGC is based on using preparations of different densities, and the SU method is based on self-migration of spermatozoa. These two methods are very gentle and have been widely applied in ART worldwide^41^,^42^. To the best of our knowledge, this is the first study to compare the telomere length in sperm cells recovered by the DGC and SU methods. In our study, the STL of the spermatozoa enriched by these both methods was longer than that in raw semen, which is consistent with previous studies that have demonstrated that either the SU or the DGS method could select sperm with a longer STL and lower DNA fragmentation^35^,^36^. Nevertheless, no significant difference was found between the STL in sperm obtained by the two procedures. We also detected differences of DNA fragmentation and ROS content, which are most closely related to STL, among samples prepared by the DGC and SU methods and raw semen. The results demonstrated that the samples prepared by either the DGC or SU method had less DNA fragmentation and lower ROS content than raw semen. However, no significant difference was found between the two approaches. These findings are similar to a previous study with 51 subjects that compared sperm recovered by the DGC and SU methods, to determine whether the method affected DNA fragmentation; no difference was found between these techniques^31^. Additionally, Ghaleno and colleagues, who obtained semen samples from 28 normozoospermic men, also found that the ROS level was not significantly different between the two methods, but the mean DFI was higher with the DGC method^33^. However, Amiri and colleagues obtained contradicting results from a study with 35 subjects, in which they found that the number of DNA-fragmented spermatozoa in samples prepared by the SU method was higher than in sperm processed with DGC^32^. Future more, one study campared the DNA fragmentation of sperm obtained by the DGC, SU and SU following DGC. There is no obvious advantage of combination than alone^31^. This inconsistency may be due to the limits of the study samples and the different techniques that were used to investigate the DNA fragmentation and ROS content as well as the different preparation processes for treating the semen samples. We also compared the sperm progressive motility among the three groups. The mean progressive motility of processed semen samples was significantly higher than in unprocessed samples. No significant difference in progressive motility was found between the two treatment groups. We concluded that both methods could be used to prepare sperm with better functional properties, which was partially illustrated by the lack of difference in pregnancy or miscarriage rates between the two techniques^20^.

We analyzed the detailed relationship between the STL and semen parameters, including the sperm DFI and ROS content in raw semen samples. The positive correlations between the STL and the total sperm count and progressive motility as well as the negative correlations between the STL and the DFI and ROS content are consistent with a previous study that indicated that the STL is associated with most of the important standard semen parameters and sperm quality^15^,^17^,^43^. And our recent study found that the semen samples of overweight/obese males had a shorter STL and increased DNA fragmentation and ROS levels^44^. The clear pathophysiological link between the STL, total sperm count and progressive motility is unknown. In fact, the STL plays a critical role in meiosis, and compromised telomere length in sperm may contribute to segregation errors, apoptosis with reduced sperm count and reduced fertility^39^,^45^. In addition, many factors commonly implicated in spermatogenesis and male infertility such as oxidative stress, infections, smoking and obesity, might be implicated in telomere shortening^46^. The pathophysiological relationship between STL, sperm DNA fragmentation and ROS is intriguing. Telomeres consist of tandem repeat sequences and telomere-associated proteins located at the ends of the chromosomes that maintain the DNA integrity. The G-rich sequences of telomeres are more sensitive to oxidative radicals that induce sperm DNA breaks than non-telomeric DNA^10^. Oxidative stress induced the accumulation of single strand breaks which lead to telomere loss during DNA replication in telomeres^47^. For instance, mild oxidative stress increased the rate of telomere shortening and over-expression of extracellular superoxide dismutase gene increased total cellular superoxide dismutase activity, decreased the intracellular peroxide content, and slowed the telomere shortening rate in human fibroblasts under standard culture conditions^47^,^48^. Additionally, dysfunctional telomeres might be recognized as a target for ROS to increase sperm DNA fragmentation and reduce telomere length. Therefore, DNA fragmentation might be both the cause and consequence of telomere shortening. Accordingly, we agree that the STL could be a parameter of sperm quality. However, unlike previous studies^15^,^43^, we found no correlation between STL and age. The reason for this outcome is likely related to the fact that we performed our study on normozoospermic men with a limited range of ages and limited samples.

In conclusion, the STL is a novel and meaningful biomarker of sperm quality and early embryo development in male reproduction. This is the first study to compare the effects of the DGC and SU sperm preparation methods on STL. And the result demonstreated that both methods can recover a sperm population with a longer STL and better DNA integrity for ART. However, the present study included only normozoospermic infertile men. The effects of the two sperm preparation methods on STL in men with oligo/asthenozoospermia should be examined in future studies.

## Materials and Methods

### Subjects

In this study, 150 subjects seeking assisted reproduction with normal standard semen parameters (WHO, 2010) were recruited from March 2015 to March 2016 at the fertility clinic of the First Affiliated Hospital of Zhengzhou University. None of the recruited patients had a Y chromosome microdeletion, karyotype anomalies, a history of parotitis, orchitis, varicocele, cryptorchidism, chemotherapy drugs or radiation treatment, systemic diseases or endocrine disorders. The study was approved by the Ethics Committee of the First Affiliated Hospital of Zhengzhou University, and each participant provided written informed consent.

All the methods used in this study were carried out in accordance with the approved guidelines.

### Sample collection and preparation

Semen samples were obtained by masturbation after 3–5 days of ejaculatory abstinence. After the samples were totally liquefied at room temperature, semen analysis was performed according to the WHO (2010) guidelines. Each sample was aliquoted into three parts: one for the SU procedure, one for DGC and one for a raw semen control without any treatment.

### Density gradient centrifugation

The DGC procedure was performed using a PureCeption^TM^ (*In Vitro* Fertilization Inc., USA) discontinuous density gradient. Briefly, one aliquot of liquefied semen was loaded onto 40% and 80% gradients (each 1.0 ml) with the 80% fraction at the bottom of a 15 ml Falcon tube (BD, USA) and then centrifuged at 300 g for 20 min at room temperature. After centrifugation, the sperm pellet was washed twice in 3 ml of pre-warmed sperm preparation medium (G-IVFTM medium [Vitro life Inc., Gothenburg, Sweden] supplemented with 10% human serum albumin [HSA, Vitro life]) and centrifuged for 5 min at 300 g. The supernatant was discarded, and the final pellet was resuspended in pre-warmed sperm preparation medium.

### Swim-up procedure

One aliquot of raw semen was gently mixed with another aliquot of pre-warmed sperm preparation medium in a 15 ml Falcon tube (BD, USA) and then centrifuged at 300 g for 10 min. The supernatant was discarded, and the pellet was resuspended in 0.5 ml of medium. Then, 0.5 ml of pre-warmed sperm preparation medium was gently layered on the sperm suspension before the sample was incubated at 37 °C for 45 min at a 45° inclination in the incubator. After incubation, 0.5 ml of supernatant was aspirated into a 15 ml centrifuge tube and washed twice with 3 ml pre-warmed IVF medium by centrifugation at 300 g for 5 min, and the pellet was finally resuspended in pre-warmed sperm preparation medium.

### Sperm chromatin dispersion test

Sperm DNA fragmentation was determined using a kit according to the manufacturer’s instructions (BRED, Life Science Technology Inc., Shenzhen, China), and the detailed steps are described in a previous study^36^. More than 500 sperm were assessed for each sample under a 100X objective on an Olympus BX51 microscope. Sperm with small nuclei or no halos were considered to contain fragmented DNA. The DNA fragmentation index (DFI) was expressed as the percentage of fragmented sperm.

### ROS production assay

The photometric nitro blue tetrazolium (NBT) test was used to evaluate ROS production of semen samples via formazan production according to a standardized protocol, which was described in our previous study^44^. Known amounts of formazan solubilized in DMSO were used to produce a standard curve of absorbance values, which was measured by a microplate reader at 630 nm (BIO-RAD, Finland). The production of ROS was expressed as mg/10^7^ spermatozoa.

### Telomere length measurement

The measurement of STL was the same as described in our previous study^15^. The genomic DNA was extracted from the sperm using the DNA Mini Kit (Qiagen 51306). The average STL of the DNA sample was analyzed using real-time PCR. Briefly, the PCR reactions were performed in 96-wellplates using the 7500 Real-Time PCR System (Applied Biosystems, USA). Each sample was run in triplicate, and a standard curve was created by serial dilutions of known amounts of reference DNA in each reaction. The relative telomere length was calculated by the telomere to single-copy gene (T/S) ratio.

### Statistical analyses

The Kolmogorov–Smirnov normality test was used to examine the normality of the data distribution. The data are presented as the means ± standard deviation or medians (ranges). One-way ANOVA and *LSD-t* tests were used to analyze the differences in parameters before and after semen preparation. A Pearson correlation or Spearman rank correlation was performed according to the normality of the variable distribution. Statistical analyses were performed by with SPSS software Version 16.0 (SPSS Inc., Chicago, IL, USA) for Windows. The level of significance was established as a two-sided *P* value of 0.05.

## Additional Information

**How to cite this article**: Zhao, F. *et al*. Semen preparation methods and sperm telomere length: density gradient centrifugation versus the swim up procedure. *Sci. Rep.* **6**, 39051; doi: 10.1038/srep39051 (2016).

**Publisher's note:** Springer Nature remains neutral with regard to jurisdictional claims in published maps and institutional affiliations.

## Figures and Tables

**Table 1:** Basic characteristics of the subjects in this study.

Parameter	Mean ± S.D./Medians (ranges)
Age (years)	31.76 ± 6.11
BMI (kg/m^2^)	23.02 ± 1.49
Duration of infertility (years)	3.50 (1.00–12.00)
Sexual abstinence (days)	4.00 (3.00–5.00)
Semen volume (ml)	2.50 ± 0.54
Sperm concentration (×10^6^/ml)	88.60 ± 29.98
Total sperm count (×10^6^)	209.11 ± 139.32
Progressive motility (%)	43.23 ± 8.17
Normal sperm morphology (%)	8.74 ± 3.98

BMI, body mass index; N = 150; values are shown as the mean ± standard deviation/medians (ranges).

**Table 2:** Correlation of human STL with sperm parameters.

Parameter	*R*	P-value^a^
Age (years)	−0.11	0.46
Duration of infertility (years)	−0.23	0.53
Sexual abstinence (days)	−0.11	0.45
Semen volume (ml)	−0.14	0.53
Sperm concentration (×10^6^/ml)	0.87	0.19
Normal sperm morphology (%)	0.26	0.12
Total sperm count (×10^6^)	0.44	0.002
Progressive motility (%)	0.42	0.003
DFI (%)	−0.44	0.002
Formazation/10^7^	−0.40	0.004

DFI, DNA fragmentation index.

^a^Pearson correlation or Spearman rank correlation according to the distribution of the variables.
